# Amiloidose Cardíaca por Transtirretina Simulando Cardiomiopatia Hipertrófica em um Paciente Idoso

**DOI:** 10.36660/abc.20200236

**Published:** 2021-04-08

**Authors:** José Pedro Alves Guimarães, Joana Trigo, Fernando Gonçalves, J. Ilídio Moreira

**Affiliations:** 1 Centro Hospitalar de Trás-os-montes e Alto Douro EPE – Cardiologia Vila Real Portugal Centro Hospitalar de Trás-os-montes e Alto Douro EPE – Cardiologia, Vila Real – Portugal

**Keywords:** Amiloidose, Cardiomiopatia Hipertrófica, Hipertensão, Insuficiência Cardíaca, Volume Sistólico, Insuficiência Renal Crônica, Ecocardiografia/métodos

A amiloidose cardíaca por transtirretina de tipo selvagem (wt-ATTR-CM) é cada vez mais reconhecida devido ao reconhecimento da prevalência crescente, avanços em métodos de diagnóstico e desenvolvimento de tratamentos eficazes.

Relatamos o caso de uma paciente de 88 anos com histórico de hipertensão, doença renal crônica (DRC), insuficiência cardíaca com fração de ejeção preservada e sem histórico familiar relevante. Ela se apresentou ao pronto-socorro com histórico de síncope, tosse produtiva, agravamento da dispneia e febre. Ausculta com sopro sistólico grau III/VI na borda esternal esquerda, ausência de sons respiratórios na base pulmonar direita e roncos bilaterais.

O eletrocardiograma revelou bloqueio atrioventricular (AV) completo; a radiografia de tórax, um edema alveolar bilateral e consolidação no pulmão direito, e os resultados analíticos foram notáveis para lesão renal aguda com hipercalemia. O bloqueio AV foi resolvido após a correção dos níveis de potássio, e ela foi internada com o diagnóstico de pneumonia adquirida na comunidade e insuficiência cardíaca descompensada.

**Figura 1:**
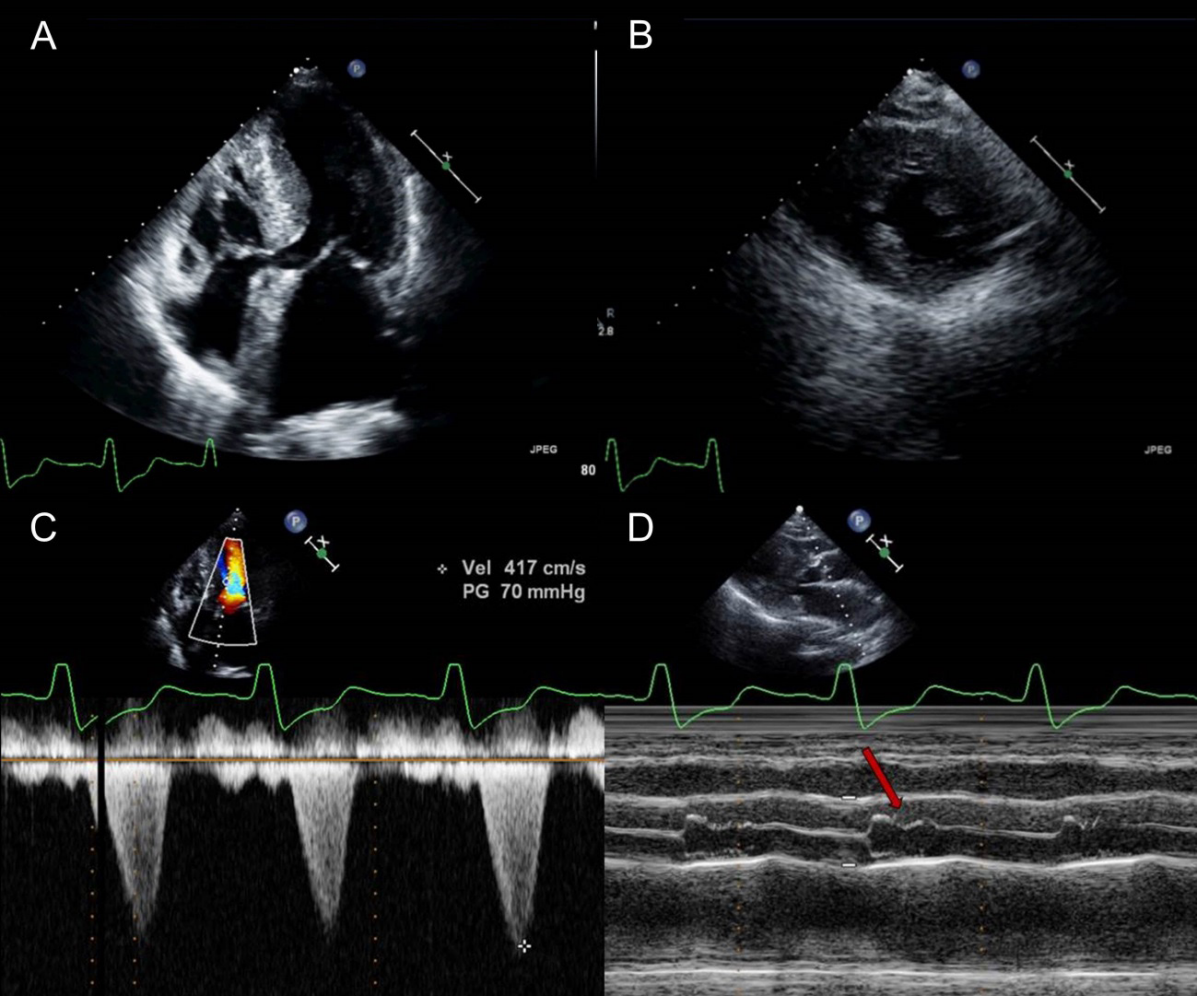
Hipertrofia assimétrica do septo (septo=19mm; parede posterior=13mm) (AB); movimento anterior sistólico da válvula mitral causando obstrução da via de saída do ventrículo esquerdo (VSVE) e encerramento mesossistólico da válvula aórtica.

**Figura 2:**
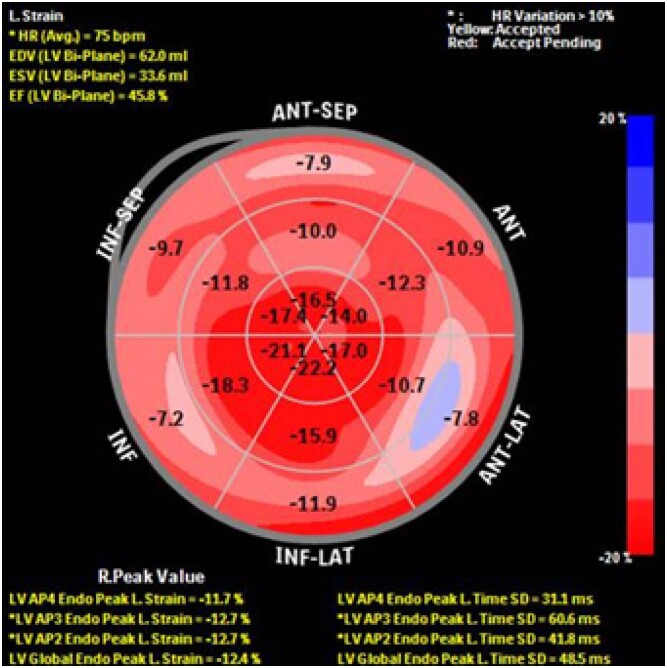
GLS reduzido (-12,4%) e preservação da deformidade miocárdica nos segmentos apicais (padrão de “apical sparing”)

O ecocardiograma transtorácico (Vídeo 1) revelou hipertrofia assimétrica do ventrículo esquerdo (Figura 1 – A e B) e movimento anterior sistólico da válvula mitral causando obstrução da via de saída do ventrículo esquerdo (VSVE), com encerramento mesossistólico da válvula aórtica (Figura 1 - C e D). Esses achados foram indicativos de cardiomiopatia hipertrófica (CMH). O VE não estava dilatado e tinha fração de ejeção preservada; sua deformação longitudinal global (GLS) foi reduzida com preservação da deformidade miocárdica nos segmentos apicais (padrão de “apical sparing”) (Figura 2). Havia insuficiência mitral moderada, insuficiência aórtica leve e a pressão sistólica da artéria pulmonar estimada era de 40 mmHg.

**Vídeo 1:**
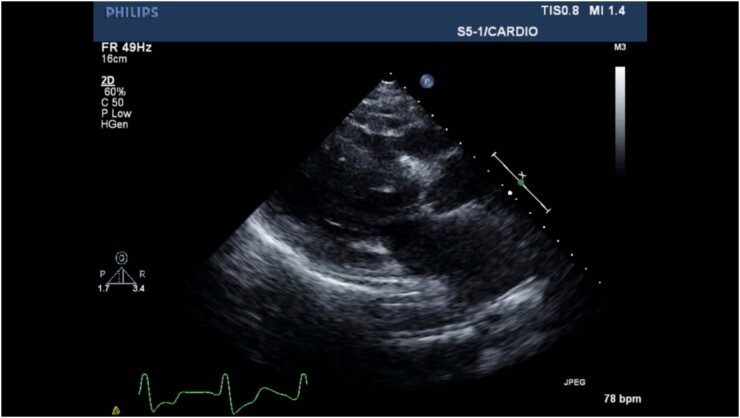
Ecocardiograma transtorácico, visão paraesternal e apical. Válvula (seta vermelha) (C-D) com gradiente intraventricular máximo de 70 mmHg. URL: http://abccardiol.org/supplementary-material/2021/11604/2020-0236-video01.mp4

**Figura 3:**
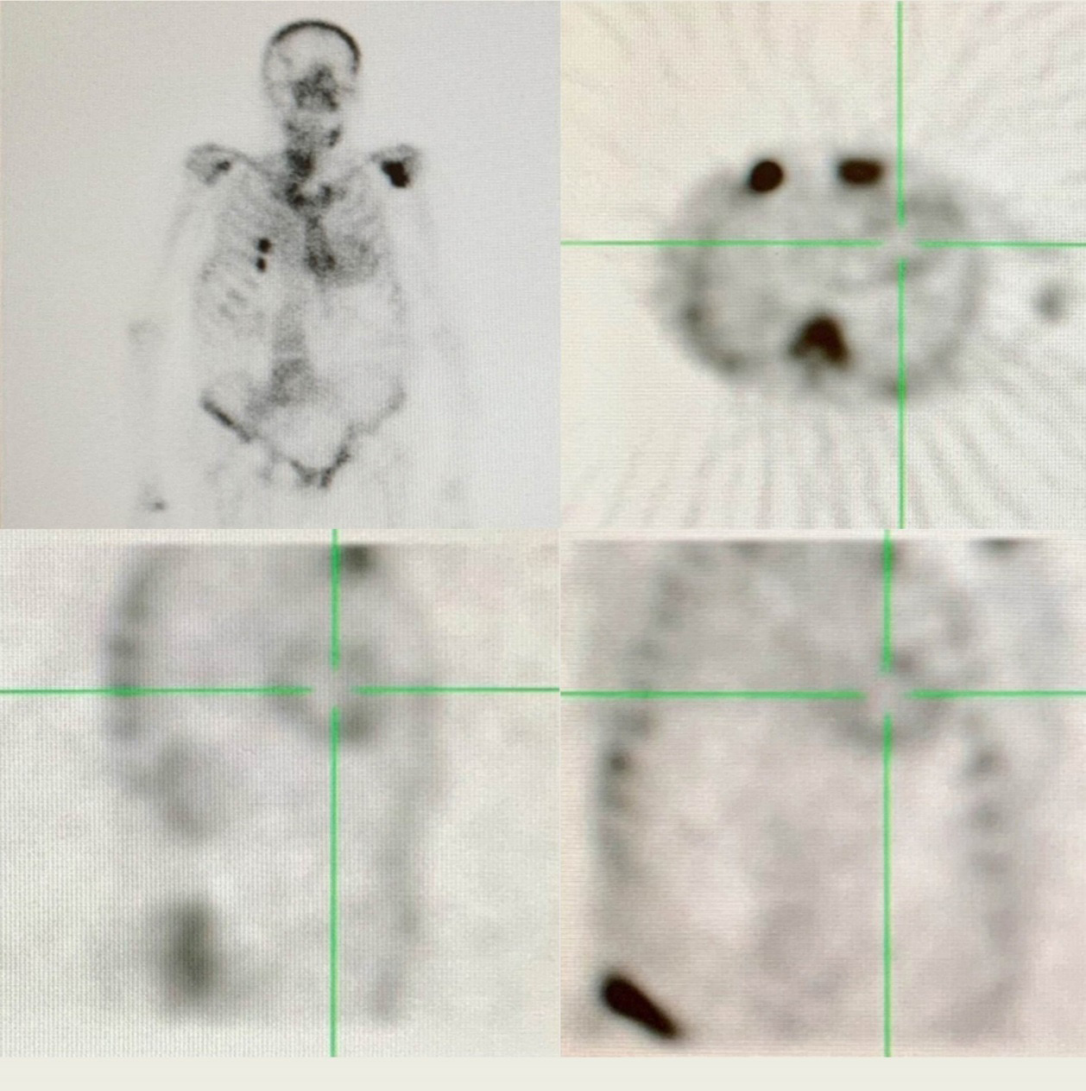
Cintilografia com DPD-99mTc mostrando captação do traçador biventricular grau II.

A cintilografia com DPD-^99m^Tc *mostrou captação difusa do traçador biventricular (grau II,* *Figura 3**), e não se encontrou* nenhuma evidência de proteína monoclonal no soro e imunofixação da urina, tampouco em ensaio de cadeia leve.

As características ecocardiográficas, a captação cardíaca com DPD-^99m^Tc e a ausência de proteína monoclonal definiram o diagnóstico de ATTR-CM.

Infelizmente, a paciente evoluiu de forma desfavorável, com superinfecção nosocomial e insuficiência cardíaca progressiva, culminando em óbito. Os resultados do teste genético de TTR foram negativos, confirmando assim o diagnóstico de wt-ATTR.

Wt-ATTR pode ser a forma mais frequente de amiloidose cardíaca,^1^ no entanto, o diagnóstico é desafiador dado o amplo espectro clínico, falta de achados “clássicos” e o fenótipo atribuído à doença cardíaca hipertensiva, estenose aórtica, ou HCM.

A ecocardiografia é o marco do diagnóstico, e o principal achado é a HVE, mas a proporção de pacientes com HVE assimétrica é alta.^2^ O *strain* é útil para o diagnóstico diferencial devido ao seu padrão diferenciado de “segmentos apicais”.^3^ Outros sinais são espessamento da válvula, espessamento do septo atrial, hipertrofia ventricular direita (HVD), dilatação biatrial, efusão pericárdica leve e aspecto em granular cintilante do miocárdio.^4^

A cintilografia com traçadores ósseos de medicina nuclear é útil para o diagnóstico não invasivo. A captação de grau II ou III na ausência de uma proteína monoclonal obteve 100% de especificidade e valor preditivo positivo em um estudo de referência.^5^ Como a amiloidose de cadeia leve é capaz de causar captação cardíaca leve e a gamopatia monoclonal de significado inderteminado é comum em pacientes mais velhos, a análise de proteína monoclonal é obrigatória. Por fim, o teste genético é necessário para distinguir entre amiloidose ATTR hereditária (ATTRh) e amiloidose de tipo selvagem (wt-ATTR).^4^

A ATTR-CM é uma causa pouco reconhecida de insuficiência cardíaca em idosos. Com o desenvolvimento de terapias eficazes, o reconhecimento e diagnóstico adequados de ATTR-CM terão um impacto terapêutico significativo.
