# Supplementary material for: Amiloidose Cardíaca por Transtirretina Simulando Cardiomiopatia Hipertrófica em um Paciente Idoso
Source: Arq Bras Cardiol. 2021 Apr 8;116(4):850–3. [Article in Portuguese] doi: 10.36660/abc.20200236 (PMC8121401; doi:10.36660/abc.20200236)
Supplement: Translation

# Translation: Transthyretin Amyloid Cardiomyopathy Mimicking Hypertrophic Cardiomyopathy in an Older Patient

**Authors:** Sora Yasri, Viroj Wiwanitkit

**Affiliations:** 1 Vila Real Portugal Centro Hospitalar de Trás-os-montes e Alto Douro EPE – Cardiologia, Vila Real – Portugal

**Keywords:** Amyloidosis, Cardiomyopathy, Hypertrophic, Hypertension, Heart Failure, Stroke, Renal, Insufficiency Chronic, Echocardiography/methods

Wild-type transthyretin amyloid cardiomyopathy (wt-ATTR-CM) is increasingly recognized due to the recognition of the increasing prevalence, advances in diagnostic methods, and the development of effective treatments.

We report the case of an 88-year-old female with history of hypertension, chronic kidney disease (CKD), heart failure with preserved ejection fraction, and no relevant family history. She presented to the emergency department with history of syncope, productive cough, worsening dyspnea, and fever. The auscultation showed a grade III/VI systolic murmur in her left sternal border, no breath sounds in the right lung base and bilateral rhonchi.

The electrocardiogram revealed a complete atrioventricular (AV) block; the chest X-ray, a bilateral alveolar edema and consolidation in the right lung, and the analytical results were remarkable for acute kidney injury with hyperkalemia. The AV block resolved after potassium levels correction, and she was admitted with the diagnosis of community acquired pneumonia and decompensated heart failure.

The transthoracic echocardiogram (Video 1) revealed asymmetric hypertrophy of the left ventricle (Figure 1 - A and B) and systolic anterior motion of the mitral valve causing obstruction of the left ventricle outflow tract (LVOT) with mid-systolic closure of the aortic valve (Figure 1 - C and D). These findings were suggestive of hypertrophic cardiomyopathy (HCM). The LV was non-dilated and had a preserved ejection fraction; her global longitudinal strain (GLS) was reduced with an apical sparing pattern (Figure 2). There was moderate mitral regurgitation, mild aortic regurgitation, and the estimated systolic pulmonary artery pressure was 40 mmHg.

**Video 1:** Transthoracic echocardiogram parasternal and apical views. Valve (red arrow) (C-D) with a maximum intraventricular gradient of 70 mmHg. URL: http://abccardiol.org/supplementary-material/2021/11604/2020-0236-video01.mp4

**Figure 1:** Asymmetric septal hypertrophy (septum=19mm; posterior wall=13mm) (A-B); systolic anterior motion of the mitral valve causing LVOT and mid-systolic closure of the aortic valve.

**Figure 2:** Reduced GLS (-12.4%) and relative apical sparing pattern.

The ^99m^Tc-DPD *scintigraphy showed diffuse biventricular tracer uptake (grade II,* *Figure 3*), and there was no evidence of a monoclonal protein in serum and urine immunofixation and in a light chain essay.

**Figure 3:** ^99m^Tc-DPD scintigraphy showing grade II biventricular tracer uptake.

The echocardiographic features, the cardiac uptake of ^99m^Tc-DPD, and the absence of a monoclonal protein defined the diagnosis of ATTR-CM.

Unfortunately, the patient had an unfavorable outcome with a nosocomial superinfection and progressive heart failure that culminated in death. The results of the TTR genetic testing were negative, thus confirming the diagnosis of wt-ATTR.

Wt-ATTR may be the most frequent form of cardiac amyloidosis,^1^ however the diagnosis is challenging given the broad clinical spectrum, lack of “classical” findings, and the phenotype attributed to hypertensive heart disease, aortic stenosis, or HCM.

Echocardiography is the diagnostic cornerstone and the main finding is LVH, but the ratio of patients with asymmetric LVH is high.^2^ Strain imaging is useful for the differential diagnosis because of its distinctive pattern of “apical sparing”.^3^ Other signs are valve thickening, atrial septal thickening, right ventricular hypertrophy, biatrial dilatation, mild pericardial effusion, and granular sparkling appearance of the myocardium.^4^

Nuclear scintigraphy using bone tracers is useful for the non-invasive diagnosis. Grade II or III uptake in the absence of a monoclonal protein had 100% specificity and positive predictive value in a landmark study.^5^ Because light-chain amyloidosis can cause mild cardiac uptake and unrelated monoclonal gammopathy is common in older patients, screening for a monoclonal protein is mandatory. Finally, genetic testing is required to distinguish between wt and hereditary-ATTR.^4^

ATTR-CM is an under-recognized cause of heart failure in older adults. With the development of effective therapies, the appropriate recognition and diagnosis of ATTR-CM will have a direct therapeutic impact.
